# A case report of an asymptomatic male child with multiple entero-enteric fistulae post multiple magnet ingestion

**DOI:** 10.1016/j.ijscr.2019.03.043

**Published:** 2019-04-09

**Authors:** Heba Taher, Ahmed Azzam, Omneya Khowailed, Mohamed Elseoudi, Muayad Shaban, Gamal Eltagy

**Affiliations:** Pediatric Surgery Department, Cairo University Specialized Pediatric Hospital, Cairo, Egypt

**Keywords:** Case report, Magnet ingestion, Asymptomatic entero-enteric fistulae, Foreign body

## Abstract

**Introduction:**

Foreign body ingestion cases are very common in children. They usually present with obvious symptoms allowing proper diagnosis and management; however, magnet ingestion can be asymptomatic. This disorder is considered uncommon as well as challenging to diagnose before complications of intestinal obstruction or peritonitis occur due to entrapment of intestinal walls in the magnetic field.

**Case presentation:**

An asymptomatic four-year-old male child was admitted to our centre after passing two bullet-shaped magnets in stool that were noticed by his mother. The child was asymptomatic.

An abdominal X-ray revealed five bullet-shaped magnets in the lower abdomen. Laboratory investigations were normal except for a mild leukocytosis. Surgery was performed with excision of seven entero-enteric fistulae and two intestinal anastomoses.

**Discussion:**

Between 2003 and 2006, the Centers for Disease Control and Prevention reported 20 complicated cases of magnet ingestion in children aged 10 months to 11 years among whom 75% had bowel perforations, and 20% suffered from generalized peritonitis. Half that number of patients required emergency laparotomy. As a result, the Consumer Product Safety Commission (CPSC) issued the first warning announcing the hazards of high-powered magnets used in children’s toys, which had been increasing exponentially. It is clear that the diagnosis of magnet ingestion is made commonly due to complications, such as peritonitis or death. However, the patient in this case was completely asymptomatic and had no complications.

**Conclusion:**

A high index of suspicion regarding complications is recommended in asymptomatic cases of suspected magnet ingestion. Avoid delaying intervention once a diagnosis has been made.

## Highlights

• Multiple magnet ingestion in children is challenging to diagnose if asymptomatic.
• Symptoms include vomiting, abdominal pain, discoloured stools or fever.
• Meticulous history, patient examination and investigations are necessary.
• Intestinal pressure necrosis and multiple fistulas can occur yet remain asymptomatic.
• Resection and anastomosis may be the sole management in such cases.

## 1 Introduction

A considerable number of toys include magnetic components that can be easily detached and then swallowed by children. These incidents may pass unnoticed as an average of 40% of foreign body ingestions are unwitnessed, and in many cases, the child may not manifest any clinical symptoms or signs [1].

Furthermore, single view X-rays may not reveal the presence of a foreign body in the patient’s gastrointestinal tract (GIT); thus, if the patient is asymptomatic, he or she may be discharged with no further management. These cases may only be noticed if the child passes a foreign body in his or her stool.

In this study, a case report of a child with asymptomatic magnet ingestion with the passage of two bullet-shaped magnets in stool and multiple intestinal fistulae is presented.

This work has been reported in accordance with the SCARE criteria [2].

## 2 Case presentation

The mother of a four-year child with no previous history of any medical conditions sought medical help at our institute because of concerns about her child’s wellbeing followed by his older brother’s report that seven of his magnet toys went missing a month earlier. In addition, she noticed that only two magnets had passed in his stools.

The child did not complain of any symptoms suggestive of foreign body ingestion, and there was no vomiting, diarrhoea, constipation or any features of abdominal pain or discomfort. In addition, his mother stated that there was no change in his eating habits or his bowel movements. On physical examination, the patient looked well and was vitally stable. There were no signs suggestive of peritonitis; his abdomen was soft and not tender or distended.

**Fig. 1:**
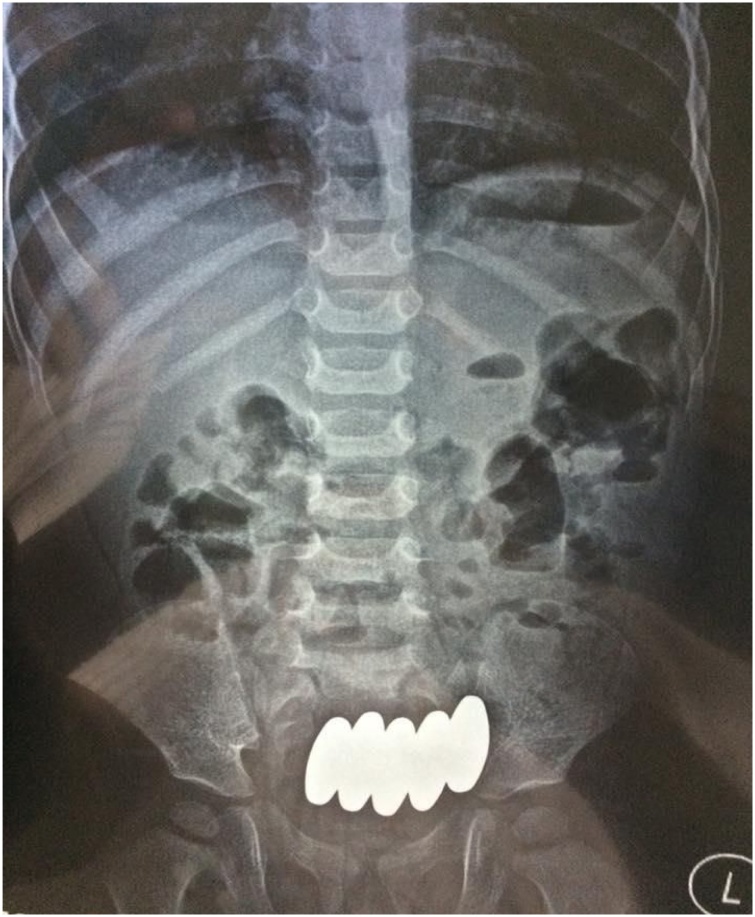
An erect abdominal X-ray showing five bullet shaped magnets in the lower abdomen with no air-fluid levels or air under the diaphragm.

Laboratory investigations were normal except for a mild leukocytosis. An erect abdominal X-ray demonstrated the presence of five bullet-shaped magnets in the lower abdomen without air-fluid levels or air under the diaphragm (Fig. 1).

**Fig. 2:**
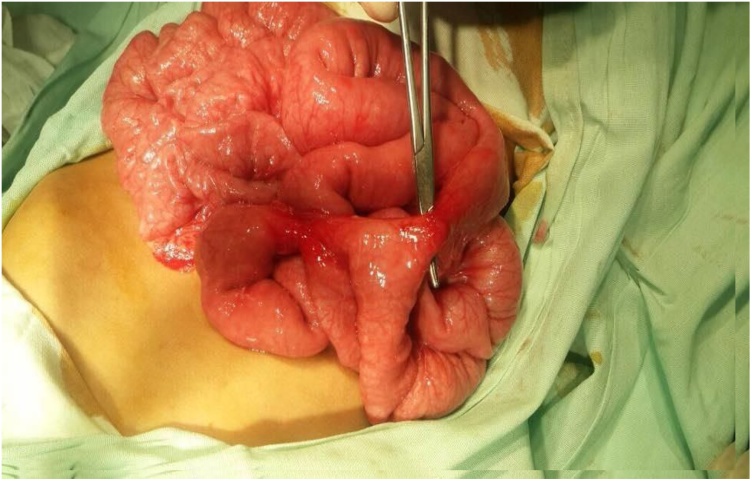
Entero-enteric fistula.

**Fig. 3:**
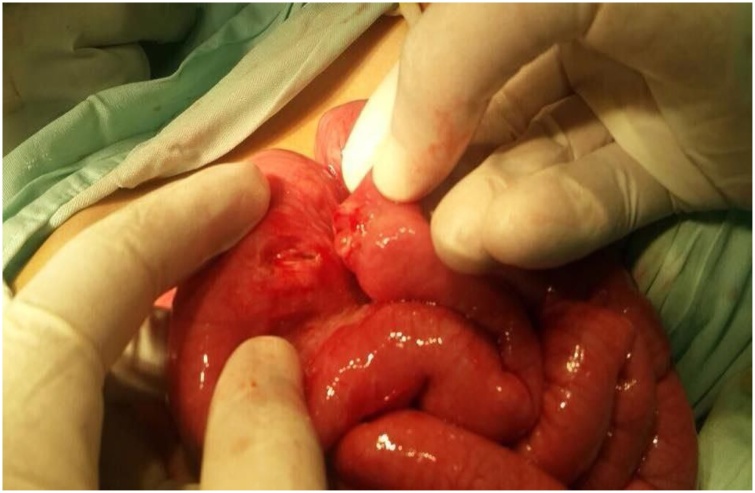
Trans-mesenteric entero-enteric fistula.

**Fig. 4:**
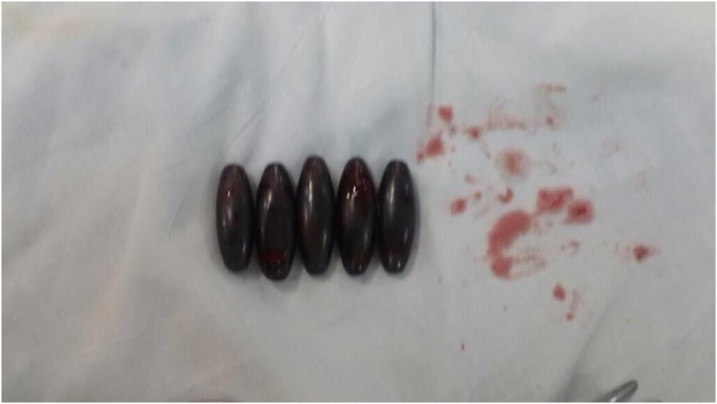
Five bullet shaped magnets retrieved from the patient’s bowel.

Because of the longstanding history of ingestion and the presence of multiple magnets in the bowel, we decided to perform exploratory abdominal surgery through a transverse supra-umbilical incision for fear of the patient developing future complications. We found seven entero-enteric fistulae, one trans-mesenteric and the remainder on the anti-mesenteric border of the bowel. Excising the fistulae yielded fourteen openings, twelve of which were close to each other along jejunal and ileal loops. We resected this part of the bowel, which was approximately 40 cm, and performed anastomosis using 4/0 Vicryl. The remaining two openings were further distal, approximately 20 cm from the ileocaecal junction, and we resected 5 cm of the bowel and performed anastomosis using Vicryl 4/0 (Fig. 2, Fig. 3). Five magnets were retrieved from the bowel (Fig. 4).

Post-operatively, the patient was placed on intravenous (IV) fluids and antibiotics. He had normal bowel function on the 2^nd^ post-operative day, and oral feeding was initiated. He was discharged on the 6^th^ post-operative day without any complications.

## 3 Discussion

Cases of multiple magnet ingestion are considered underreported, as only less than 55 cases have been identified in the English literature over the past decade [3].

The earliest case from Japan in 1995 was complicated by bowel perforation [4].

Between 2003 and 2006, the Centers for Disease Control and Prevention published a report of 20 complicated cases of magnet ingestion in children aged 10 months to 11 years among whom 75% had bowel perforations, and 20% suffered from generalized peritonitis [5]. The alarming death of a 20-month-old child who swallowed a number of magnets and 33 other magnet ingestion cases were reported. Half that number required emergency laparotomy to save the infants’ lives [6,7]. As a result, the Consumer Product Safety Commission (CPSC) issued the first warning of the hazards of high-powered magnets used in children’s toys, which had been increasing exponentially. These magnets can become detached and swallowed by infants during exploration and result in intestinal injuries or even death [6,8,9]. Another study showed that both gender and age play a role in magnet ingestion cases, as most affected patients were males, and approximately 50% were younger than 5 years [10].

It is clear that the diagnosis of magnet ingestion is made commonly due to complications, such as peritonitis or death [11,12]. In contrast, this patient was completely asymptomatic and had no complications. It has been proven that the ingestion of one magnet is unlikely to cause significant harm, as the magnet will probably be eliminated spontaneously. However, if multiple magnets are ingested or a magnet is ingested with other metallic material, the two will be powerful enough to attract each other through the walls of the intestine, causing pressure necrosis and resulting in multiple perforations and fistulae and eventually peritonitis.

These cases are difficult to diagnose because their symptoms are quite similar to typical flu symptoms or other gastrointestinal illnesses, such as vomiting, abdominal pain, diarrhoea and fever [13]. However, this was not the case with our patient, as the child had no complaints and presented because of his mother’s concern that he had two bullet-shaped magnets in his stool and due to his older brother’s report of missing magnets a month previously.

Although conservative treatment is sufficient in some cases of single magnet ingestion, magnets can still cause serious morbidities depending on their size, shape and magnetic field; therefore, endoscopic or surgical intervention is occasionally required [14]. If more than one magnet has been ingested, then endoscopic removal must be performed without delay unless the magnets have travelled beyond the pylorus. Once magnets have passed the pylorus, some authors prefer prompt surgical intervention even if the patient is asymptomatic to assess the vitality of the bowels [14].

On the other hand, some authors advocate the policy of delaying surgery until signs of peritoneal complications occur [15]. Various therapeutic modalities involve the intentional use of two magnets placed together to create a nonsurgical intestinal anastomosis [15].

In our case, the inadvertently formed jejunoileal fistula allowed the passage of 2 magnets without causing peritonitis; therefore, the remaining 5 magnets could have passed in a similar fashion, and the treatment would have seemed successful. However, blind loop syndrome is a known complication following side-to-side bypass of the intestine [16]. Moreover, there are few studies on the long-term sequelae of small bowel-to-small bowel fistulae formed during infancy [15].

Therefore, expectant management alone in the absence of abdominal complications is not always justified [15].

Most abdominal symptoms are known to occur between 1 and 7 days after ingestion of multiple magnets [15]. The earliest documented asymptomatic entero-enteric fistula was discovered upon laparotomy 24 h post-magnet ingestion, and the reason for laparotomy was that the family was not sure about number of magnets swallowed despite the passage of 3 magnets; furthermore, one magnet that the doctors could not confirm if it was a single magnet or 2 adherent magnets persisted on X-ray; ultimately, 2 magnets were confirmed to be causing necrosis and fistula formation at laparotomy [15].

In the current case, 30 days passed after the ingestion, and only 2 magnets out of 7 passed. The remaining 5 magnets were obviously attached to each other on an abdominal X-ray (Fig. 1). Therefore, surgical exploration and removal of the remaining magnets to prevent potential complications, such as intestinal fistulae formation, and/or to address such complications was deemed the safest decision.

## 4 Conclusion

Assessment of any patient with suspected of foreign body ingestion requires careful history taking from both parents as well as siblings of the patient about any missing toys, magnets or any stool abnormalities. This evaluation should be followed by a meticulous physical examination. If magnet ingestion is suspected, prompt investigations, such as multiple view X-rays, must not be delayed, and MRI must be avoided [17]. If the presence of magnets in the patient’s bowels is confirmed by the X-rays, immediate intervention with endoscopic removal is advised if the magnets are located proximal to the pylorus or surgical exploration if the magnets are located distal to the pylorus [14]. In cases of perforated bowel suggested by the presence of air under the diaphragm, surgical intervention in the form of exploratory surgery, retrieval of the magnets, resection of the fistulae and non-viable loops followed by anastomosis is recommended.

In some cases, the absence of symptoms does not guarantee the absence of complications, such as fistula formation. Due to paucity of evidence on the long-term sequelae of small bowel-to-small bowel fistulae formation during infancy, surgical intervention should not be delayed [15].

## Conflicts of interest

We declare no conflict of interest.

## Sources of funding

Not applicable.

## Ethical approval

Ethical approval has been exempted by our institution because this is a case report this is a case report and no new studies or new techniques were carried out.

## Consent

Written informed consent was obtained from the parents of the patient, since the patient is a minor, for publication of this case report and accompanying images. A copy of the written consent is available for review by the Editor-in-Chief of this journal on request.

## Author’s contribution

Ahmed Azzam: Operated on patient drafting the manuscript.

Omnia: Drafting the manuscript and literature search.

Mohamed Elseoudi: Operated on the patient, drafting of the manuscript.

Heba Taher: Clinical Supervision and guidance, drafting manuscript.

Muayad Shaban: Literature search.

Gamal Eltagy: Clinical supervision and consultation.

## Registration of research studies

Not applicable.

## Guarantor

Heba Taher.

## Provenance and peer review

Not commissioned, externally peer-reviewed.
